# "Drunk or Dying" Cases

**Published:** 1903-03-14

**Authors:** 


### Editor's Letter-Box.

**[Our Correspondents are reminded that prolixity is a great bar to publication, and that brevity of style and conciseness of statement greatly facilitate early insertion.]**

### "DRUNK OR DYING" CASES.

The Hon. Sydney Holland writes under date 9th inst.:—"It is quite impossible for anyone with any self-respect to reply to a question put to him in such a peculiarly offensive language as you have used towards me. 'Is it too much to expect,' you write, 'that Mr. Holland will answer this straightforward question in a straightforward manner?' I never mind any of the 'give-and-take' of a controversy, and will answer any question put civilly, but it is simply intolerable to so frame a question as to indicate that a *straightforward* answer from me would be a surprise."

